# Effect of nanoprocessing on the physicochemical properties of bovine, porcine, chicken, and rabbit bone powders

**DOI:** 10.1002/fsn3.2312

**Published:** 2021-05-18

**Authors:** Xue Li, Zhifei He, Jingbing Xu, Ling Zhang, Yexing Liang, Shixiong Yang, Zefu Wang, Dong Zhang, Feihu Gao, Hongjun Li

**Affiliations:** ^1^ College of Food Science Southwest University Chongqing China; ^2^ Agricultural Product Processing Institute Chongqing Academy of Agricultural Science Chongqing China; ^3^ Chongqing Engineering Research Center of Regional Food Chongqing China; ^4^ Chongqing Institute for Food and Drug Control Chongqing China

**Keywords:** animal bone, characteristics, nanoprocessing, rabbit

## Abstract

The chemical composition and hardness of bovine bone, porcine bone, chicken bone, and rabbit bone were compared, as well as the influence of nanoprocessing on the physicochemical characteristics of these bone powders. A series of nanofabrication processes led to an increase in bone minerals and the loss of protein and fat. The hardness of softened bovine bone was still the largest, whereas chicken and rabbit bones were relatively soft. There were no significant differences in the functional groups between nanoscale bone powders. Overall, nanomachining significantly reduced and homogenized the bone particle size and improved the color and release rate of calcium ions of bone powders at the same time; these effects were different for several bones. Nanoscale rabbit bone had higher comminution efficiency, as well as satisfactory nutritional value, color, and product yield, which supports its strong development potential.

## Graphical abstract

This study mainly described the application of nanoprocessing in common mammalian bones (bovine and porcine), common poultry bone (chicken), and new material rabbit bone. The effects of nanoprocessing on the physicochemical properties of rabbit bone and other three common bones were compared. The excellent properties of nanoscaled rabbit bone (NRB), which support its great development potential.

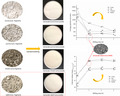

## 1 INTRODUCTION

In the Neolithic age, people were known to eat animal bones to supplement calcium (Vieugué et al., 2015). Today, people can also buy healthy foods made of animal bone powders to enhance bone density. For example, FINE brand fish bone calcium powder and ZOVLA brand fish bone calcium tablet are produced in Japan, and CELESSE ANGEL brand yak marrow strong bone dust is produced in China. To improve the taste, these bone powders are usually manufactured into tablets or mixed with such ingredients as rice flour, sesame powder, or milk powder. Generally, bone use has been limited in the field of food study due to its coarse and grainy texture, and bone is still mainly used as animal feed or plant fertilizer because of its low price (Buckley et al., 2012; Genisel et al., 2012). To improve the added value of animal bones, some of them are used for intensive processing, including extraction of bone protein (Dong et al., 2014; Song et al., 2016) and bone oil (Choi et al., 2016; Hagura et al., 2002), and preparation of natural hydroxyapatite (Heidari et al., 2018; Nam et al., 2019), gelatin (Li et al., 2013; Wang et al., 2016), and essence (You et al., 2020). However, the utilization of bone in these processes is limited, and more comprehensive use of bone still needs further study.

In recent years, some researchers have studied the application of nanotechnology in bone processing. Through ball milling, dynamic high‐pressure microfluidization, and other physical means (Sha et al., 2018; Yin et al., ,^2015^, ^2016^; Zhang et al., 2016), whole bone is crushed into nanoscale particles (100 to 1,000 nm), which make full use of bone nutrients and improve the properties of bone particles. Nanorefining improves the palatability, water‐holding capacity, calcium solubility, and bioavailability of fish bone powder (Yin et al., 2015); adding nanoscale bone powder into surimi and other foods also improves the nutritive value and properties of the gel (Li et al., 2018; Yin et al., 2014). Therefore, nanotechnology has potential applications in animal bone processing.

Fish processing makes use of nanotechnology and provides value. Other bones, such as beef, pork, chicken, and rabbit bones, could also utilize this technology. Rabbit meat is becoming increasingly popular worldwide as a functional meat product due to its special nutritional characteristics (Zotte & Szendrő, 2011). The total output of rabbit meat in the world was 1.4 million tons in 2018, whereas that of China was 0.87 million tons, which continued to be at the forefront of the world (Food & Agriculture Organization of the United Nations, Statistics Division, 2020). With the development of the rabbit meat industry, the amount of rabbit bone and other by‐products has also increased. Bone is a by‐product with high nutritional value in meat processing, and its utilization can bring economic benefits and reduce environmental pressure. However, most rabbit meat processing enterprises do not use rabbit bones; therefore, its processing and utilization will be imminent. The bones of different species have different chemical compositions, hierarchical structures, and mechanical properties. The differences in these raw materials may affect the nanoprocessing and product characteristics. To conduct the high‐value deep processing of animal bone and explore the nanoprocessing characteristics of rabbit bones and those of other animals, this study compared the chemical composition of rabbit bone, common mammalian bones (bovine and porcine), and common poultry bone (chicken), as well as contrasted the effects of nanoprocessing and differences in physicochemical properties between nanoscale bovine bone (NBB), nanoscale porcine bone (NPB), nanoscale chicken bone (NCB), and nanoscale rabbit bone (NRB).

## 2 MATERIALS AND METHODS

### 2.1 Materials

The age and breed of each animal group in the experiment were similar; 4 males and 4 females made up the groups of cattle (24 months old, Xianan) and pigs (6 months old, Taihu × Duroc × York crossbreed), and 30 males and 30 females made up the groups of chickens (45 days old, Rose 308) and rabbits (70 days old, Hyla). Animal femurs were selected as representative raw materials, and whole femurs from bovines, porcines, chickens, and rabbits were removed from the legs of 76 chilled carcasses (purchased from a local slaughterhouse) and cleaned of muscle, fat, and tendons by hand immediately. The average weight of each rabbit bone was 9.61 ± 0.34 g, chicken bone was 18.13 ± 3.39 g, porcine bone was 397.40 ± 14.40 g, and bovine bone was 1,056.20 ± 147.11 g.

All femurs were vacuum‐packaged and then frozen at −21°C for 48 hr. A total of 6 femurs were taken from each group for texture analysis, and the remaining femurs were ground through a bone crusher (PG‐230, Yongchuang Food Machinery Co., Ltd., Zhengzhou, China) with a 10‐mm screen twice to produce particles of approximate size (5 mm). For composition analyses, 100 g of bone shards were further crushed and blended by a grinder (BJ‐800A, Baijie Technology Co., Ltd., Hangzhou, China) and then packed in vacuum and stored at −60°C, and the other bone shards were directly vacuum‐packaged and stored at −21°C for nanoscale bone powder (NBP) processing. Unless otherwise specified, all chemicals used were analytical grade or higher and purchased from Ke Long Chemical Co. Ltd, Chengdu, China.

### 2.2 NBB, NPB, NCB, and NRB preparation

All NBPs were produced according to a common process, which was described by Li et al. (2018) with small modifications. A total of 300 g of frozen bone shards in each group were thawed in flowing tap water at room temperature and then rinsed 3 times to remove blood and fat. These bone pieces were submerged in water at a ratio of 1:1 (w/v) and heated at 120°C for 4 hr by using an automatic high‐pressure cooker (LDZM‐80KCS, Shenan Medical Instrument Factory, Shanghai, China) followed by rinsing 3 times again. Subsequently, the remaining bone residues were enzymatically hydrolyzed (enzyme dosage of 5,000 U/g, substrate concentration of 15%) with papain (100,000 U/g, Solarbio Technology Co., Ltd., Beijing, China) at 55°C for 2 hr and inactivated by boiling for 10 min. The upper liquid was drained off, and the bone residues were dried at 50°C for 12 hr in a constant temperature air blower drying oven (DHG‐9053J, Sanfa Scientific Instrument Co., Ltd, Shanghai, China). Dry bone residues were crushed by the grinder, and these bone meals were further degreased according to a method described by Buckley et al. (2012). The bone meals were lipid‐extracted with 100% hexane at a ratio of 1:1 (w/v) twice (constantly mixed for 15 min and 2 hr). The lipid‐extracted samples were then centrifuged at 2,042 *g*, and the supernatants were discarded. The red plasma protein on the surface of the sediments was scraped off by a medicine spoon, and the remaining sediments were dried to constant weight at 50°C in the drying oven. Next, these dried defatted bone meal particles were crushed to micron scale by a vibrating microgrinder (SYFM‐8II, Songyue Machinery Co., Ltd., Jinan, China) with a vibration amplitude of 5 mm and time of 15 min.

Finally, NBB, NPB, NCB, and NRB were obtained by ball milling the micron‐scale bone powders (MBPs) using a planetary ball mill (XQM‐0.4, Tianchuang Powder Technology Co., Ltd., Changsha, China). Except for the ball milling time, the ball milling process was consistent with Li et al. (2018). The samples of bone powders were collected after grinding for 0, 1, 3, and 5 hr. At each sampling time, approximately 15 g samples were randomly gathered from each treatment (3 replicates), and a total of 48 individual powders were collected for the measurement of relevant indicators. After milling for 5 hr, NBB, NPB, NCB, and NRB, which are collectively referred to as NBPs, were prepared, and the samples milled for 0 hr (MBPs) were used as the control group.

### 2.3 Chemical composition analysis

The moisture, crude protein, crude fat, and ash contents of the fresh bones and NBPs were determined by standard A.O.A.C. methods. Elements were determined by inductively coupled plasma‐atomic emission spectrometry (iCAP 6000, Thermo Fisher, USA), as described by Ersoy and Özeren (2009). Amino acid composition analysis was carried out by an amino acid analyzer (S433D, Sykam, Germany), which was equipped with a lithium system for the determination of 35 kinds of amino acids. The sample pretreatment, solution preparation, and procedures were performed as outlined by Sykam Corp. The fatty acid composition of the fresh bones was estimated by gas chromatography (7890A, Agilent, USA) using a 60 m × 0.25 mm × 0.25 μm capillary column (DB‐FFAP, Agilent, USA) in accordance with a method described by Tume et al. (2010), and the fatty acid methyl esters were identified by comparison of retention times with authentic standards (CDAA‐252795‐MIX, Anpel Laboratory Technology Co., Ltd., Shanghai, China).

### 2.4 Vibration spectroscopy

According to Boutinguiza et al. (2012) and Novák et al. (2012), the chemical structure of NBPs was recorded on a Fourier transform infrared spectrometer (Spectrum 100, PerkinElmer, USA), and the spectra were collected in the wavenumber range of 4,000 to 400 cm^−1^. Additionally, Raman spectra were recorded on a Raman spectrometer (DXR2, Thermo Fisher, USA) equipped with a laser source (785 nm) with an energy of 15.0 mW and a grating of 400 lines mm^−1^; each sample was exposed 40 times for 5 s, and the spectra were collected in the wavenumber range of 3,300 to 50 cm^−1^.

### 2.5 Bone hardness measurement

A total of 6 frozen femurs from each group were thawed in flowing tap water at room temperature and then softened by an automatic high‐pressure cooker at 120°C for 4 hr before hardness detection. After cooking, 4 cm bone cadres were taken from the center of the femur using a knife and then dried to a constant weight at 50°C. Hardness (g) of cooked bone was determined by a texture analyzer (CT3, Brookfield, USA) with a blade probe (TA 7). The bone cadres were placed horizontally on the platform to measure vertical fracture failure stress. A compression test was performed with the equipment set as trigger force of 1 g, test speed of 1.0 mm/s, and compression deformation of 50%.

### 2.6 Observation of micromorphology

The morphology of bone particles was observed using a scanning electron microscope (ProX, Phenom, Netherlands) with a magnification of 7,000 and an acceleration voltage of 10.0 kV. The dried bone powders were dispersed in thin film on the double‐sided adhesive of a bronze platform and sputter‐coated with gold before observation.

### 2.7 Measurement of particle size

The particle size was expressed by the hydrodynamic diameter. The particle size of MBPs in the micrometer range (1 to 1,000 μm) was measured by Mastersizer 2000 (Malvern, U.K.) according to Ullah et al. (2017). The size distribution of NBPs particles was analyzed by a Zetasizer Nano ZS90 analyzer (ZEN3690, Malvern, UK), as described by Yin et al. (2015) with some modification. Bone particles were diluted to 1.0 g/100 g using ultrapure water, and the dispersant (sodium hexametaphosphate at 0.2 g/100 g) was added (constantly mixed for 2 min), and the samples were treated with ultrasonic dispersing (KQ‐600DB, Ultrasonic Instrument Co., Ltd., Kunshan, China) for 15 min at room temperature (25°C).

### 2.8 Analysis of calcium release

The calcium release of bone particles was assayed by the method of Yin et al. (2016) and Zhang et al. (2016) with minor modifications. Bone powder (0.25 g) was suspended in 25 ml digestive juice (aqueous solution of pH 2 adjusted by 5 mol/L HCl) with 2 mg/ml pepsin (3,500 U/g protein; Solarbio, Beijing, China). The suspension was incubated at 37°C for 3 hr in a constant temperature shaker at 100 rpm (MaxQ 4000, Thermo Fisher, USA). After extraction, the samples were centrifuged at 4,000 *g* for 30 min (5810R, Eppendorf, Germany). The supernatant was filtered and diluted to 250 ml with ultrapure water. Finally, the content of calcium was determined by ICP‐OES. The calcium release was calculated according to the following formula:

(1) $$\text{Calcium}\;\text{release}\left(\text{mg}/g\right)=S/M$$

where *S* is the calcium content in the supernatant (in mg) and *M* is the weight of bone powder in the suspension (in g).

### 2.9 Color measurement

Objective color of bone particles was measured using a colorimeter (CM‐2300d, Konica Minolta, Japan) with CIE lightness (L*), redness/greenness (a*), and yellowness/blueness (b*) color scale. Whiteness (*W*) was investigated using a whiteness tester (XT‐48BN, Yante Technology Co., Ltd., Hangzhou, China) with ISO brightness (R_457_). Measurements were standardized with a white calibration plate, and the surface of each sample was evaluated at 6 random locations.

### 2.10 Product yield analysis

Product yield (PY) was evaluated by dividing the weight of NBPs by the weight of raw bone and expressed as percentage according to Shackelford et al. (1994), PY1 was calculated as a proportion of fresh bone weight, and PY2 was calculated as a proportion of dry bone weight.

### 2.11 Statistical analysis

All analyses were performed at least in triplicate, and the data are presented as the mean ± standard deviation. Differences between means were determined utilizing one‐way analysis of variance (ANOVA) using SPSS software, Version 19.0 (IBM Corp., USA). A *p*‐value <.05 was established to be a significant difference, and a *p*‐value <.01 was confirmed to be an extremely significant difference.

## 3 RESULTS AND DISCUSSION

### 3.1 Chemical composition

The chemical composition comparison results of different kinds of bone are shown in Table 1 and Table 2. In fresh bone, percent fat and dry matter on a fresh weight basis; crude protein, ash, some minerals, and amino acid contents on a dry‐fat‐free basis; and fatty acid composition in total fatty acids are gathered in Table 1. The data show that bones are rich in minerals, protein, and fat, which have high nutritional value. The data for bovine bone, porcine bone, and chicken bone are in accordance with most of the reports by Field et al. (1974). The chemical composition of different kinds of animal bone was significantly different (*p* < .05).

**TABLE 1:** Chemical composition of fresh bone

Measure	Cattle	Pig	Chicken	Rabbit
Composition of fresh bone				
Fat (%)	20.87 ± 0.36^b^	28.14 ± 0.82^a^	8.77 ± 0.28^c^	8.34 ± 0.16^c^
Dry matter (%)	76.55 ± 0.29^b^	79.74 ± 0.32^a^	43.64 ± 0.25^d^	52.17 ± 0.14^c^
Composition of dry‐fat‐free bone				
Protein (%)	35.81 ± 1.20^c^	32.47 ± 1.85^c^	48.45 ± 1.25^a^	41.47 ± 0.98^b^
Ash (%)	64.1 ± 0.24^b^	65.34 ± 0.25^a^	50.40 ± 0.04^d^	58.98 ± 0.17^c^
Ca (mg/g)	296.54 ± 2.43^a^	271.90 ± 1.21^b^	220.65 ± 1.40^d^	250.82 ± 2.63^c^
Mg (mg/g)	5.92 ± 0.19^a^	4.52 ± 0.26^c^	4.51 ± 0.24^c^	5.01 ± 0.22^b^
Zn (mg/kg)	86.42 ± 1.02^d^	165.96 ± 1.19^c^	183.08 ± 2.16^b^	225.83 ± 1.04^a^
Gly (%)	6.74 ± 0.22^a^	6.58 ± 0.18^a^	5.99 ± 0.01^b^	5.76 ± 0.03^b^
Pro (%)	4.23 ± 0.31^a^	3.87 ± 0.02^ab^	3.64 ± 0.02^b^	3.43 ± 0.07^b^
Hyp (%)	3.77 ± 0.27^a^	3.44 ± 0.05^a^	2.59 ± 0.17^b^	2.68 ± 0.01^b^
Glu (%)	3.70 ± 0.13^c^	3.53 ± 0.05^c^	5.22 ± 0.15^a^	4.48 ± 0.11^b^
Ala (%)	2.95 ± 0.22^b^	2.79 ± 0.04^b^	3.34 ± 0.06^a^	2.89 ± 0.02^b^
Arg (%)	2.74 ± 0.16^bc^	2.56 ± 0.06^c^	3.12 ± 0.09^a^	2.84 ± 0.03^ab^
Asp (%)	1.49 ± 0.13^c^	1.40 ± 0.03^c^	2.32 ± 0.09^a^	1.93 ± 0.01^b^
Lys (%)	1.33 ± 0.07^c^	1.19 ± 0.08^c^	1.97 ± 0.14^a^	1.63 ± 0.02^b^
Leu (%)	1.25 ± 0.11^c^	1.15 ± 0.02^c^	2.38 ± 0.12^a^	2.01 ± 0.02^b^
Ser (%)	1.19 ± 0.09^b^	1.10 ± 0.01^b^	1.54 ± 0.06^a^	1.47 ± 0.01^a^
Phe (%)	0.89 ± 0.09^c^	0.88 ± 0.01^c^	1.71 ± 0.07^a^	1.37 ± 0.01^b^
Val (%)	0.88 ± 0.08^b^	0.91 ± 0.02^b^	1.60 ± 0.07^a^	1.47 ± 0.04^a^
Thr (%)	0.81 ± 0.10^c^	0.69 ± 0.01^c^	1.48 ± 0.12^a^	1.25 ± 0.02^b^
Ile (%)	0.51 ± 0.05^c^	0.42 ± 0.01^c^	1.05 ± 0.05^a^	0.74 ± 0.00^b^
Tyr (%)	0.28 ± 0.04^c^	0.27 ± 0.00^c^	0.78 ± 0.04^a^	0.63 ± 0.00^b^
His (%)	0.20 ± 0.02^c^	0.20 ± 0.01^c^	0.54 ± 0.03^a^	0.47 ± 0.00^b^
Met (%)	0.08 ± 0.06^b^	0.15 ± 0.05^b^	0.48 ± 0.01^a^	0.29 ± 0.15^ab^
Cys (%)	0.04 ± 0.01^b^	0.05 ± 0.00^b^	0.31 ± 0.05^a^	0.27 ± 0.01^a^
Tau (%)	0.04 ± 0.00^c^	0.05 ± 0.00^c^	0.15 ± 0.01^a^	0.09 ± 0.00^b^
EAA	5.74 ± 0.55^c^	5.38 ± 0.20^c^	10.66 ± 0.27^a^	8.76 ± 0.21^b^
NEAA	27.38 ± 1.77^ab^	25.84 ± 0.45^b^	29.53 ± 0.41^a^	26.93 ± 0.09^ab^
Fatty acids (g/100 g total fatty acids)				
C14:0	3.89 ± 0.11^a^	0.94 ± 0.00^c^	0.00 ± 0.00^d^	2.10 ± 0.01^b^
C14:1	0.44 ± 0.19^a^	0.00 ± 0.00^b^	0.00 ± 0.00^b^	0.00 ± 0.00^b^
C15:0	0.97 ± 0.03^a^	0.00 ± 0.00^c^	0.00 ± 0.00^c^	0.69 ± 0.00^b^
C15:1	0.38 ± 0.01^a^	0.00 ± 0.00^c^	0.00 ± 0.00^c^	0.22 ± 0.00^b^
C16:0	25.36 ± 0.62^c^	21.31 ± 0.03^d^	30.93 ± 0.01^a^	29.68 ± 0.02^b^
C16:1	3.13 ± 0.08^b^	1.30 ± 0.01^d^	6.20 ± 0.09^a^	2.30 ± 0.01^c^
C17:0	1.35 ± 0.03^a^	0.37 ± 0.01^c^	0.00 ± 0.00^d^	0.96 ± 0.00^b^
C17:1	0.74 ± 0.02^a^	0.19 ± 0.00^c^	0.00 ± 0.00^d^	0.25 ± 0.00^b^
C18:0	17.55 ± 0.39^a^	10.78 ± 0.01^b^	11.27 ± 0.10^b^	7.99 ± 0.01^c^
C18:1	40.78 ± 0.94^b^	37.97 ± 0.05^c^	43.78 ± 0.08^a^	27.14 ± 0.00^d^
C18:2n6	2.16 ± 0.05^d^	21.70 ± 0.02^b^	7.83 ± 0.08^c^	26.38 ± 0.02^a^
C18:3n3	0.43 ± 0.01^c^	0.86 ± 0.01^b^	0.00 ± 0.00^d^	1.18 ± 0.00^a^
C20:0	0.14 ± 0.00^a^	0.00 ± 0.00^b^	0.00 ± 0.00^b^	0.00 ± 0.00^b^
C20:1	0.13 ± 0.00^c^	1.85 ± 0.00^a^	0.00 ± 0.00^d^	0.61 ± 0.00^b^
C20:2	0.00 ± 0.00^c^	1.92 ± 0.00^a^	0.00 ± 0.00^c^	0.49 ± 0.01^b^
C20:3n6	0.00 ± 0.00^b^	0.10 ± 0.14^a^	0.00 ± 0.00^b^	0.00 ± 0.00^b^
C20:4n6	0.00 ± 0.00^b^	0.34 ± 0.00^a^	0.00 ± 0.00^b^	0.00 ± 0.00^b^
C20:3n3	0.00 ± 0.00^b^	0.38 ± 0.00^a^	0.00 ± 0.00^b^	0.00 ± 0.00^b^
*n*−6	2.16 ± 0.05^d^	22.13 ± 0.11^b^	7.83 ± 0.08^c^	26.38 ± 0.02^a^
*n*−3	0.43 ± 0.01^c^	1.24 ± 0.01^a^	0.00 ± 0.00^d^	1.18 ± 0.00^b^
SFAs	49.26 ± 1.18^a^	33.40 ± 0.04^c^	42.20 ± 0.09^b^	41.43 ± 0.03^b^
MUFAs	47.92 ± 1.25^b^	41.31 ± 0.06^c^	49.97 ± 0.01^a^	30.52 ± 0.00^d^
PUFAs	2.82 ± 0.07^d^	25.29 ± 0.10^b^	7.83 ± 0.08^c^	28.05 ± 0.03^a^
UFAs	50.74 ± 1.18^c^	66.60 ± 0.04^a^	57.80 ± 0.09^b^	58.57 ± 0.03^b^
UFAs/SFAs	1.03 ± 0.05^c^	1.99 ± 0.00^a^	1.37 ± 0.01^b^	1.41 ± 0.00^b^

Note

Different lowercase (a–d) superscripts in the same row indicate significant differences (*p* < .05).

**TABLE 2:** Chemical composition of nanoscale bone powders

Measure	NBB	NPB	NCB	NRB
Proximate (%)				
Fat	1.95 ± 0.22^b^	2.31 ± 0.13^a^	2.02 ± 0.11^b^	2.30 ± 0.16^a^
Moisture	3.92 ± 0.16^a^	3.44 ± 0.14^a^	3.51 ± 0.21^a^	3.70 ± 0.25^a^
Protein	10.18 ± 0.25^b^	9.13 ± 0.35^b^	14.05 ± 0.47^a^	9.90 ± 0.58^b^
Ash	84.00 ± 0.42^a^	84.84 ± 1.28^a^	80.53 ± 0.95^b^	83.52 ± 0.55^a^
Minerals				
Ca (mg/g)	309.03 ± 2.18^c^	313.44 ± 2.17^b^	285.83 ± 2.55^d^	326.00 ± 2.29^a^
Mg (mg/g)	7.44 ± 0.18^a^	6.09 ± 0.24^b^	7.28 ± 0.14^a^	7.26 ± 0.23^a^
Zn (mg/kg)	94.13 ± 2.55^c^	187.56 ± 2.13^b^	241.55 ± 2.83^a^	259.02 ± 3.02^a^
Amino acid (%)				
Gly	1.60 ± 0.06^a^	1.30 ± 0.04^b^	1.07 ± 0.02^c^	1.02 ± 0.10^c^
Pro	0.97 ± 0.03^a^	0.89 ± 0.11^ab^	0.80 ± 0.01^c^	0.86 ± 0.04^ab^
Hyp	0.81 ± 0.04^a^	0.73 ± 0.14^a^	0.41 ± 0.07^b^	0.46 ± 0.04^b^
Glu	0.89 ± 0.04^c^	0.85 ± 0.02^c^	1.58 ± 0.01^a^	1.13 ± 0.12^b^
Ala	0.65 ± 0.02^bc^	0.57 ± 0.02^c^	0.79 ± 0.02^a^	0.71 ± 0.07^ab^
Arg	0.65 ± 0.05^b^	0.60 ± 0.02^b^	0.91 ± 0.00^a^	0.46 ± 0.03^c^
Asp	0.38 ± 0.02^c^	0.37 ± 0.02^c^	0.78 ± 0.01^a^	0.46 ± 0.05^b^
Lys	0.22 ± 0.03^a^	0.20 ± 0.01^ab^	0.15 ± 0.00^b^	0.24 ± 0.01^a^
Leu	0.31 ± 0.01^c^	0.32 ± 0.03^c^	0.86 ± 0.01^a^	0.49 ± 0.05^b^
Ser	0.28 ± 0.01^c^	0.26 ± 0.01^c^	0.52 ± 0.01^a^	0.33 ± 0.03^b^
Phe	0.26 ± 0.00^c^	0.27 ± 0.00^c^	0.71 ± 0.01^a^	0.34 ± 0.03^b^
Val	0.21 ± 0.01^c^	0.22 ± 0.00^c^	0.55 ± 0.01^a^	0.35 ± 0.04^b^
Thr	0.18 ± 0.01^c^	0.17 ± 0.00^c^	0.51 ± 0.05^a^	0.27 ± 0.03^b^
Ile	0.12 ± 0.00^b^	0.12 ± 0.02^b^	0.38 ± 0.01^a^	0.16 ± 0.01^b^
Tyr	0.08 ± 0.00^c^	0.09 ± 0.00^c^	0.35 ± 0.00^a^	0.18 ± 0.03^b^
His	0.07 ± 0.00^c^	0.09 ± 0.00^c^	0.29 ± 0.01^a^	0.13 ± 0.01^b^
Met	0.02 ± 0.02^b^	0.04 ± 0.01^b^	0.18 ± 0.06^a^	0.09 ± 0.05^ab^
Cys	0.01 ± 0.00^c^	0.02 ± 0.01^c^	0.08 ± 0.01^a^	0.04 ± 0.01^b^
Tau	0.02 ± 0.00^a^	0.02 ± 0.00^a^	0.02 ± 0.00^a^	0.02 ± 0.00^a^
EAA	1.31 ± 0.03^c^	1.34 ± 0.06^c^	3.33 ± 0.16^a^	1.81 ± 0.13^b^
NEAA	6.46 ± 0.27^b^	5.85 ± 0.39^b^	7.65 ± 0.15^a^	5.94 ± 0.54^b^

Note

Different lowercase (a–d) superscripts in the same row indicate significant differences (*p* < .05).

Porcine bone had the highest fat content (28.14 ± 0.82%) followed by bovine bone (20.87 ± 0.36%), whereas chicken bone (8.77 ± 0.28%) and rabbit bone (8.34 ± 0.16%) had the lowest fat content. The low‐fat characteristics of chicken bone and rabbit bone would reduce the burden of defatting bone meals in the later stage, whereas the high‐fat characteristics of porcine bone and bovine bone could provide a good source for animal fat extraction. Fatty acid composition analysis shows that the content of saturated fatty acids (SFAs) in bovine bone oil was the highest, which was mainly composed of C16:0, C18:0, C14:0, and C17:0, as reported by Mello et al. (1976). In chicken bone oil, the content of monounsaturated fatty acids (MUFAs) was the highest, which was mainly composed of C18:1 and C16:1 and is similar to that described by Dolegowska et al. (2006). The content of polyunsaturated fatty acids (PUFAs) in rabbit bone oil was the highest, which was mainly composed of long chain n‐6 and n‐3 PUFAs (C18:2n6 and C18:3n3) and is consistent with the representation of Zotte and Szendrő (2011) in rabbit meats. This may be related to the feeding diets of rabbit. The fatty acid composition of muscle foods from monogastrics can be easily changed by diet, and the PUFA content of rabbit carcasses could be increased by supplementing their diets with alfalfa grass (Zotte & Szendrő, 2011). Porcine bone oil had the highest content of unsaturated fatty acids (UFAs), which were mainly composed of C18:1, C18:2n6, C20:2, and C20:1, and this is similar to the findings of Wood et al. (2008) in pork adipose tissue. In addition, porcine bone oil had the highest content of long chain n‐3 PUFA, which intake is much more important than the n‐6/n‐3 ratio. Although the fat in bone would be removed in the subsequent nanoprocessing for storage period and powder dispersion, the utilization of bone oil after solvent recovery is worthy of further study. Due to the high moisture content, the dry matter content of chicken bone (43.64 ± 0.25%) and rabbit bone (52.17 ± 0.14%) was significantly lower (*p* < .05) than that of porcine bone (79.74 ± 0.32%) and bovine bone (76.55 ± 0.29%), which may have a great impact on the yield of bone meal.

To determine the theoretical value of the chemical composition of NBPs, most of the data are presented in Table 1 on a dry‐fat‐free basis. Excluding the influence of moisture and fat, chicken bone and rabbit bone have higher protein contents (>41%) and total amino acids (>35%). The main component of the protein in animal bone is type I collagen, which contains Gly‐Pro‐Hyp characteristic sequences (Kittiphattanabawon et al., 2005). Therefore, glycine, proline, and hydroxyproline are shown as the main amino acids in bone protein in Table 1. The collagen content of porcine bone and bovine bone was higher, whereas chicken bone and rabbit bone had more advantages in the content of essential amino acids (EAA). On the other hand, the ash content of porcine bone and bovine bone was higher (>64%), which was consistent with the trend of calcium. The magnesium content of bovine bone and rabbit bone was higher. Bone salts are mainly composed of calcium hydrogen phosphate (CaHPO_4_) and hydroxyapatite (Ca_10_(PO_4_)_6_(OH)_2_), and Ca^2+^, Mg^2+^, and other ions are adsorbed on the surface of these two salts (Venkatesan et al., 2015). It is worth mentioning that rabbit bone had not only high protein content (41.47 ± 0.98%) but also satisfactory ash content (58.98 ± 0.17%). In addition to 250.82 ± 2.63 mg/g calcium concentration and 5.01 ± 0.22 mg/g magnesium concentration, rabbit bone also contained the highest content of zinc (225.83 ± 1.04 mg/kg).

After a series of nanoprocesses, such as high‐pressure cooking, enzymatic hydrolysis, degreasing, decolorization, drying, and grinding, fresh animal bones were produced into NBPs, and the chemical compositions are presented in Table 2. The chemical composition of animal bone changed greatly. Because drying removes a large amount of water from animal bones, the moisture content in NBPs was approximately 3%. Most of the fat in NBPs was removed by hot water and n‐hexane such that the fat content in NBPs was reduced to approximately 2%. High‐temperature cooking and water washing decolorization led to the loss of collagen, hemoglobin, and other proteins, and the loss rate of protein was approximately 71% to 76%. The loss of protein also reduced the content of most amino acids in animal bones, but chicken bone still retained the highest protein content (14.05 ± 0.47%) and total amino acid content (10.98 ± 0.16%). At the same time, the ash, calcium, magnesium, and zinc contents in animal bone were increased due to the concentration effect of water, fat, and some protein removal. In general, NBPs prepared from animal bones retained many minerals (ash content 80.53% to 84.84%), proteins (9.13% to 14.05%), and amino acids (7.19% to 10.98%), which are natural resources with high nutritional value. It may be due to high ash content and the loss of more protein in processing, and NRB contained the highest content of inorganic salt (83.52 ± 0.55%), calcium content (326.00 ± 2.29 mg/g), magnesium content (7.26 ± 0.23 mg/g), and zinc content (259.02 ± 3.02 mg/kg), which give rabbit bone an advantage as a nutritional supplement.

### 3.2 Chemical structure

**FIGURE 1:**
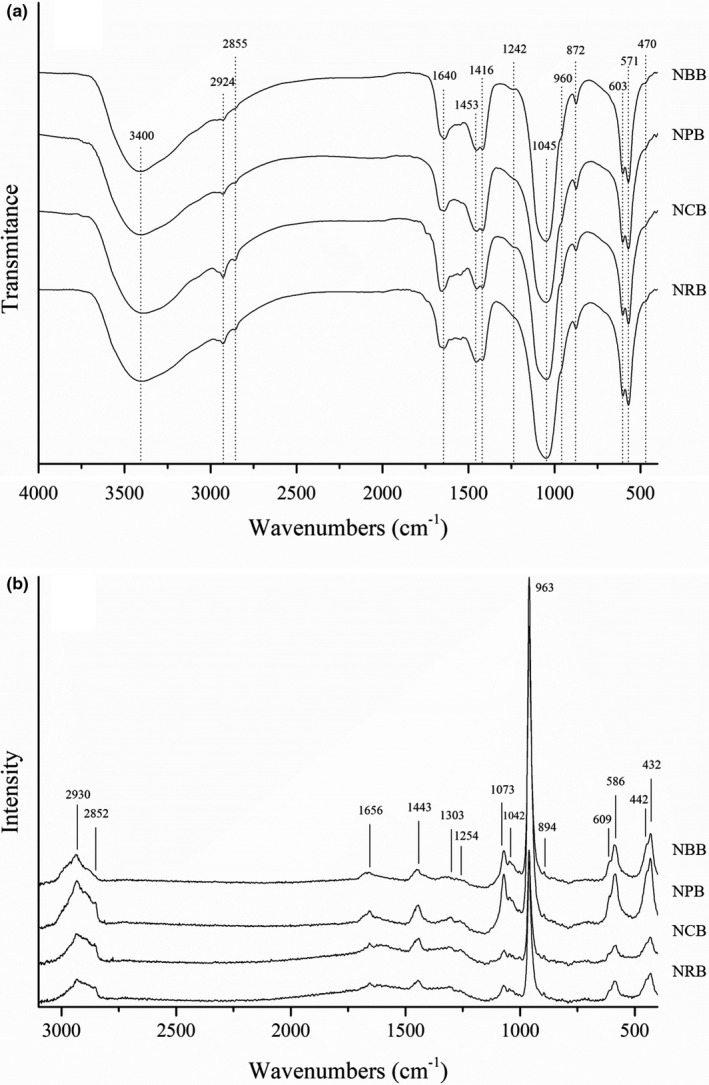
FT‐IR (A) and Raman (B) spectra of nanoscale bone powders

FT‐IR and Raman spectra of NBPs are shown in Figure 1. The wide IR band near 3,400 cm^−1^ represented the OH stretching vibration of hydroxyls and residual water in the samples (Venkatesan et al., 2015). IR bands at 2,924 and 2,855 cm^−1^ and corresponding peaks in the Raman spectra indicated CH stretching vibrations of bone organic matter (Boutinguiza et al., 2012). There were three regions representing amides in the IR/Raman spectrum at 1,640 cm^−1^ (Amide I), 1565–1520 cm^−1^ (Amide II), and 1303–1242 cm^−1^ (Amide III), which were related directly to the structure of the protein in NBPs. Amide I is the sensitive region of the protein secondary structure, whose absorption band is almost entirely from the contribution of C = O stretch vibrations; the amide II band is related to the NH bending vibration and CN stretching vibration of the protein; and the amide III band is the characteristic absorption peak of collagen, which is related to the triple helix structure (Cebi et al., 2016; Plepis et al., 1996). The peaks of the amide III band in NBPs were weak, which might be related to the degradation of collagen after cooking. The region of carbonate ion vibrations had two Raman bands (1,443 and 894 cm^−1^) and three IR bands (1,453, 1,416, and 872 cm^−1^) (D'Elía et al., 2013). The obvious phosphate group peaks in NBPs were observed by Raman and IR spectra, and the characteristic peaks representing phosphate in the Raman spectrum were more elaborate. The Raman spectra were dominated by a very strong peak at 963 cm/cm (v1), the medium intensity peaks presented at 432 cm^−1^ (v2), 586 cm^−1^ (v4), and 1,073 cm^−1^ (v3), and the lower intensity peaks appeared at 442 cm^−1^ (v2), 609 cm^−1^ (v4), and 1,042 cm^−1^ (v3), whose distributions were similar to the structures of synthetic and natural hydroxyapatite (Heidari et al., 2018; Nam et al., 2019).

The waveforms of several NBPs were similar with high overlap, which indicates that their chemical structures and compositions are similar. The characteristic peaks showed the presence of hydroxyapatite and proteins such as collagen. This is consistent with the results of the chemical composition analysis of NPBs. The main components of NBPs are minerals such as hydroxyapatite and residual proteins such as collagen. The tiny differences in the NBPs spectra might be due to the degradation degree of collagen and other proteins from several animal bones, and the substitution of the phosphate group site in the hydroxyapatite lattice was replaced by carbonate ions (D'Elía et al., 2013; Venkatesan et al., 2015).

### 3.3 Bone hardness

**FIGURE 2:**
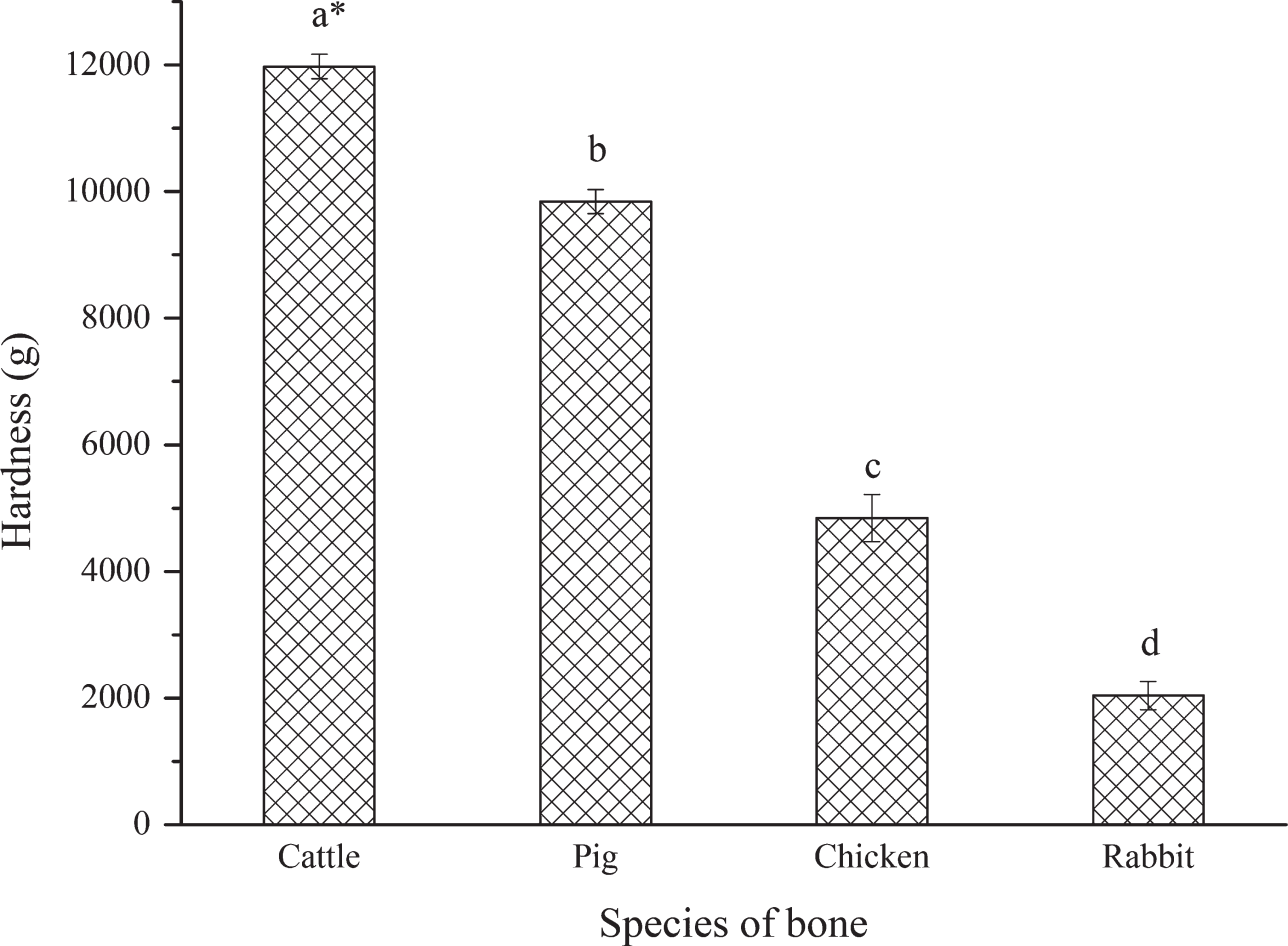
Hardness of bone softened by high‐pressure cooking. *denotes that the instrument load was exceeded; values with different lowercase letters (a–d) are significantly different (*p* < .05)

Raw animal bones are very hard, and it is difficult to break the bone directly, so they need to be softened before they can be refined. In this study, conventional high‐pressure cooking was used to soften the bones, and the hardness of several softened bones is illustrated in Figure 2. After cooking (120°C for 4 hr), the rabbit bone and chicken bone softened obviously, but the hardness of bovine bone was overloaded (the maximum load of instrument was 10 kg), so the hardness of bovine bone was the highest, followed by porcine bone, chicken bone, and rabbit bone (*p* < .05). Hydroxyapatite crystals and the collagen network structure are the main explanations of bone hardness (Yin et al., 2015). High‐temperature pretreatment leads to bone collagen denaturation and degradation of its natural triple helix structure, which results in bone fracture failure stress reduction. The difference in bone hardness may be related to the residual collagen and mineral salt thickness. Cattle and pigs have large bone and thick sclerotin layers and retain more collagen under the same cooking conditions (confirmed by the Gly‐Pro‐Hyp content analysis results shown in Table 2), which gives them high hardness. The difficulty of bone comminution depends on the hardness of bone, so it could be inferred that rabbit bone and chicken bone are easier to soften and break, whereas porcine and bovine bones are more difficult, especially bovine bone, which is the most difficult to fracture.

### 3.4 Particle size and microstructure

**FIGURE 3:**
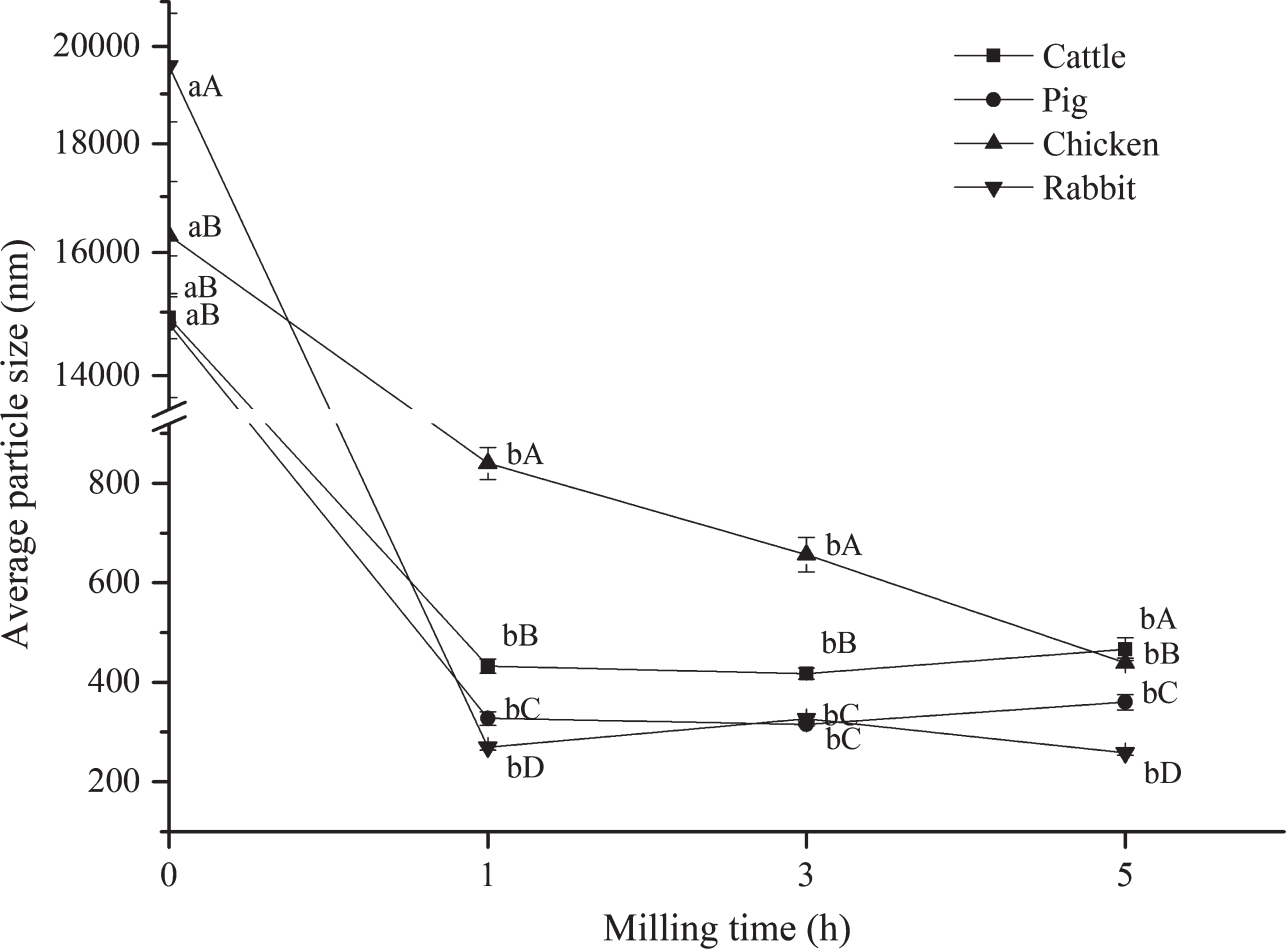
Changes in the average particle size of several kinds of bone under different milling times. Values with different lowercase letters (a–b) indicate a significant difference between milling times for the same species (*p* < .05) and uppercase letters (A–D) indicate a significant difference between species for the same milling time (*p* < .05)

**FIGURE 4:**
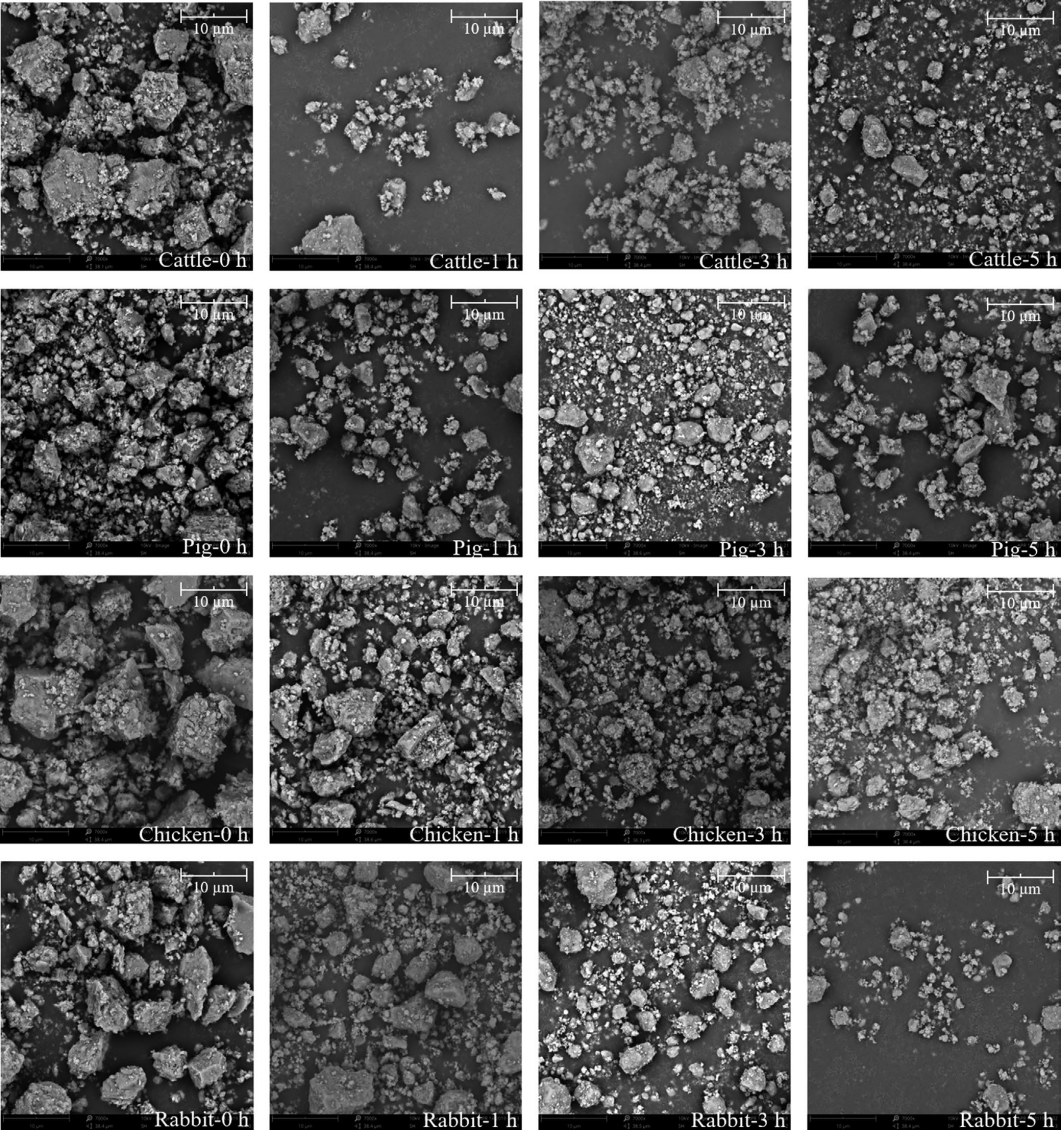
Microstructure of several kinds of bone under different milling times

The changes in the average particle size of various bones dispersed in water under different milling times are presented in Figure 3. After only 1 hr of milling, the average particle size of each group decreased significantly (*p* < .05) and reached the nanometer range (269–840 nm). The average particle size of rabbit bone powder was the smallest (269.87 ± 5.76 nm), followed by porcine bone powder (327.50 ± 13.65 nm), bovine bone powder (432.43 ± 13.97 nm), and chicken bone powder (840.23 ± 32.32 nm) (*p* < .05). The particle size of chicken bone was larger, which may be related to the more protein agglomerates adhered on its surface (Figure 4). With the extension of milling time, the particle size of chicken bone decreased, the particle size of bovine and porcine bones first decreased and then increased, while the particle size of rabbit bone increased first and then decreased. Although these changes were not significant (*p* > .05) compared with milling for 0 hr, it can be seen from the microscope pictures (Figure 4) that several kinds of bone changed from irregular polygonal to spherical, and the particles became more uniform. The particle size did not continue to decrease or even increase during testing, which may be related to the grinding limit of the equipment on the raw materials and agglomeration between small particles due to the van der Waals force (Malde et al., 2010). This effect is consistent with the research results of Yin et al. (2016) in ball milling of fish bone meal. After grinding for 5 hr, the particle size of bone from smallest to largest was NRB, NPB, NCB, and NBB (*p* < .05), which was consistent with the trend of bone hardness except for chicken bone.

### 3.5 Calcium ion release

**FIGURE 5:**
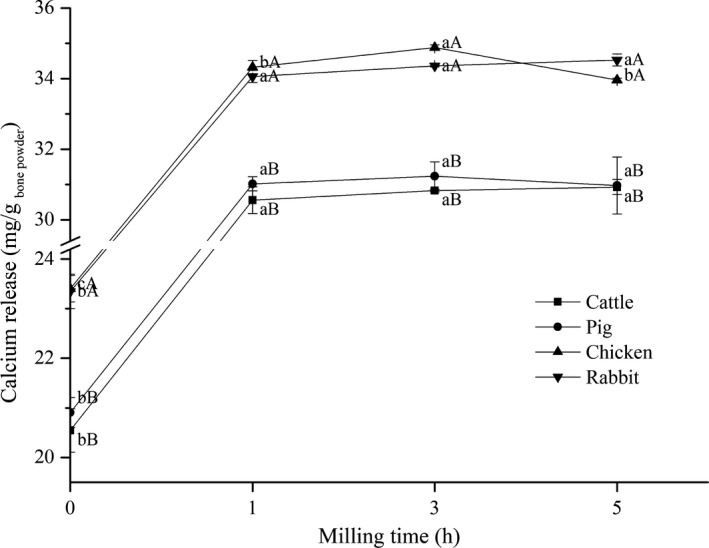
Calcium release of several kinds of bone under different milling times. Values with different lowercase letters (a–b) indicate a significant difference between milling times for the same species (*p* < .05) and uppercase letters (A–B) indicate a significant difference between species for the same milling time (*p* < .05)

Figure 5 shows that after 1 hr of ball milling, the particle size of bone powder reached the nanoscale, and the solubility of calcium ions in various bone powders significantly increased (*p* < .05). This may be because ball milling increases the specific surface area of bone powder and destroys the structure of hydroxyapatite crystals and collagen networks, thus facilitating the release of calcium ions from bone powder (Zhang et al., 2016). However, the solubility of calcium ions increased with grinding, but the difference was not significant (*p* > .05), which may be related to the limit of ball milling on bone powder. The solubility of calcium ions in rabbit bone and chicken bone was significantly higher than that in porcine and bovine bone (*p* < .05), which should be caused by the greater degree of bone fragmentation, which was unanimous with the trend of bone hardness.

### 3.6 Commercial characteristics

**FIGURE 6:**
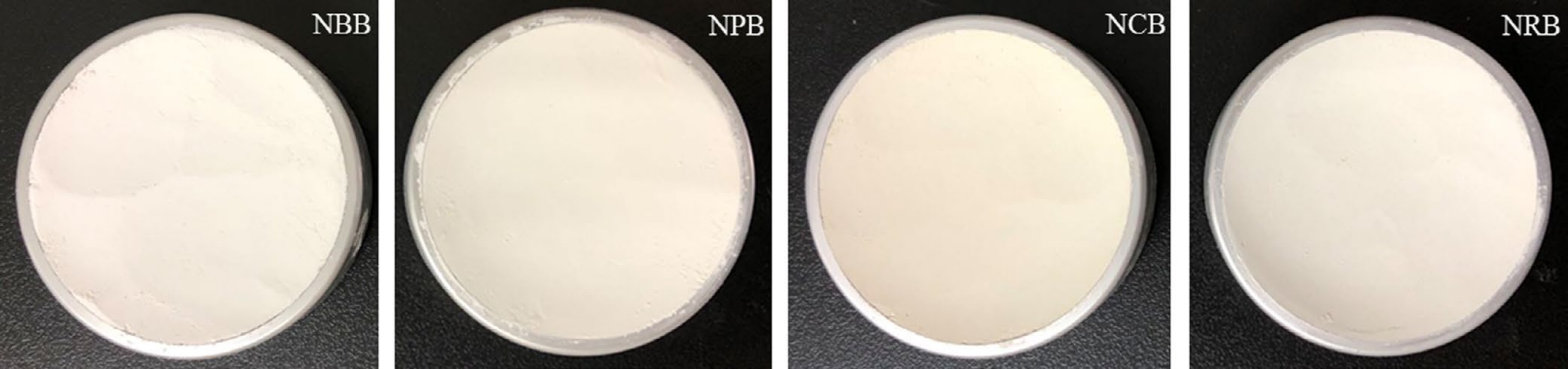
Physical photographs of nanoscale bone powders

Color is the most direct factor to attract consumers. As shown in Table 3, bone meal color was significantly affected by nanofabrication. The whiteness of bone powders increased (*p* < .01); the L* values of bone powders increased and the b* values decreased; and these changes were very significant (*p* < .01) except for bovine bone. The a* value of porcine bone and rabbit bone increased, and the a* value of chicken bone decreased. The whiteness and brightness of bone powders from highest to lowest were NBB, NPB, NRB, and NCB, and the color of NCB was slightly yellow, as shown in Figure 6. The yellow and red color may be related to the more residual hemoglobin in bone. The color of bone powder is mainly composed of white hydroxyapatite and a small amount of reddish‐brown hemoglobin. After nanotreatment, the majority of hydroxyapatite particles decreased and evenly dispersed in the powder, which might cover the color of hemoglobin. Therefore, the bone powders mainly presented the color of hydroxyapatite, in which whiteness and brightness were improved and the color was developing toward blue and green. However, further refinement likely increased the dispersion of hemoglobin in the powder, which increased the a* value of NPB and NRB. On the whole, it can be seen from Figure 6 that NBB, NPB, and NRB showed satisfactory white color, which could lay a foundation for the addition of bone powders in other foods.

**TABLE 3:** Color of micron‐scale bone powders and nanoscale bone powders

	MBPs	NBPs		MBPs	NBPs
*W*			*a* *		
Cattle	78.26 ± 0.03^a^	80.72 ± 0.03^a**^	Cattle	−0.58 ± 0.02^d^	−0.61 ± 0.10^b^
Pig	68.41 ± 0.03^b^	70.82 ± 0.05^b**^	Pig	−0.78 ± 0.03^b^	−0.69 ± 0.02^ab**^
Chicken	41.48 ± 0.03^d^	55.53 ± 0.03^d**^	Chicken	−0.65 ± 0.02^c**^	−0.76 ± 0.01^a^
Rabbit	57.77 ± 0.08^c^	61.48 ± 0.03^c**^	Rabbit	−0.87 ± 0.02^a^	−0.78 ± 0.03^a*^
*L* *			*b* *		
Cattle	95.57 ± 0.24^a^	96.01 ± 0.45^a^	Cattle	3.25 ± 0.06^d^	2.86 ± 0.68^d^
Pig	93.62 ± 0.02^b^	94.60 ± 0.07^b**^	Pig	5.06 ± 0.07^c**^	4.19 ± 0.10^c^
Chicken	86.09 ± 0.07^d^	89.34 ± 0.05^d**^	Chicken	10.75 ± 0.17^a**^	8.38 ± 0.04^a^
Rabbit	89.75 ± 0.17^c^	91.28 ± 0.08^c**^	Rabbit	6.99 ± 0.12^b**^	5.68 ± 0.04^b^

Note

Values with different lowercase superscript letters (a–d) in the same column are significantly different for the same color index (*p* < .05).

* Superscripts indicate a significant difference in the same row (*p* < .05), while ** indicates an extremely significant difference (*p* < .01).

**FIGURE 7:**
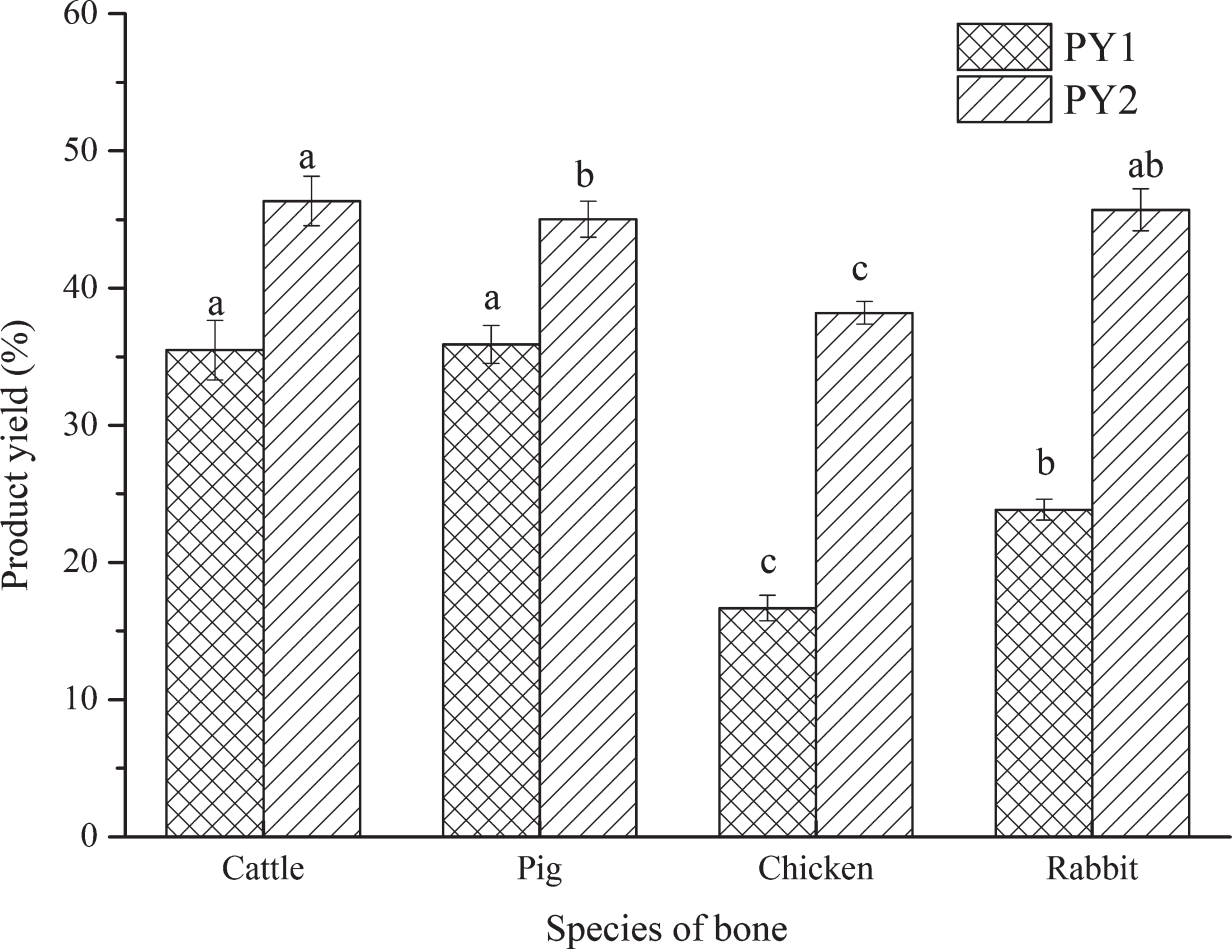
Product yield of different bones. Different lowercase letters (a–c) in the same column indicate significant differences (*p* < .05)

The product yield is closely related to the economic benefit of production. As shown in Figure 7, the PY1 of bovine and porcine bone was higher, followed by rabbit bone and chicken bone (*p* < .05). This decrease in chicken and rabbit bones may be due to their higher moisture content. Nevertheless, dried bones are sometimes directly collected as raw material in industrial production. Without considering the factor of moisture, the PY2 of rabbit bone was close to that of bovine and porcine bones, although the yield of chicken bone was still lower (*p* < .05), which should be caused by more protein loss in chicken bone processing. In addition, from the perspective of bone hardness, that is, the crushing time, the requirements, and maintenance of crushing equipment, rabbit bone and chicken bone have their own advantages. From the perspective of animal reproduction speed, the speed of supplying raw rabbit bone cannot be underestimated.

## 4 CONCLUSION

The studied four kinds of animal bones have different physicochemical characteristics. In actual production, bone raw materials can be selected according to their chemical composition and processing properties. After a series of nanoprocessing steps, the mineral content of bones increased, and the protein and fat content decreased. The main chemical substances and structures of the NBPs were similar, but the proportions were different. Overall, the bones still retained many minerals and proteins, especially calcium, so NBPs can be used as a good source of natural calcium. Nanoprocessing reduced the size and unified bone particles, and the calcium solubility and color of bone powders were also improved. Nanotechnology is worth popularizing in the processing of animal bone meal.

It is worth mentioning that rabbit bone has high nutritive value and low processing intensity. The NRB was rich in protein (9.90 ± 0.58%), calcium (326.00 ± 2.29 mg/g), magnesium (7.26 ± 0.23 mg/g), and zinc (259.02 ± 3.02 mg/kg). Moreover, NRB had the smallest particle size (258.18 ± 5.04 nm), good crushing efficiency, high calcium ion release rate, light color, and considerable yield. As an undeveloped halal material with a high reproduction speed, rabbit bone has certain application prospects.

## CONFLICT OF INTEREST

The authors declare no conflict of interest in this work.

## AUTHOR CONTRIBUTIONS

**Xue Li:** Conceptualization (lead); Data curation (lead); Formal analysis (lead); Methodology (lead); Writing‐original draft (lead). **Zhifei He:** Investigation (lead); Methodology (supporting); Project administration (supporting). **Jingbing Xu:** Methodology (supporting). **Ling Zhang:** Investigation (supporting). **Yexing Liang:** Methodology (supporting). **Shixiong Yang:** Methodology (supporting). **Zefu Wang:** Writing‐review & editing (lead). **Dong Zhang:** Writing‐review & editing (supporting). **Feihu Gao:** Investigation (supporting); Resources (supporting). **Hongjun Li:** Funding acquisition (lead); Project administration (lead).

## ETHICAL APPROVAL

This article does not contain any studies with human or animal subjects.

## Data Availability

Data openly available in a public repository that issues datasets with DOIs.
